# Are sub‐alpine species' seedling emergence and establishment in the alpine limited by climate or biotic interactions?

**DOI:** 10.1002/ece3.11009

**Published:** 2024-02-13

**Authors:** Ingrid J. Dahle, Ragnhild Gya, Joachim P. Töpper, Vigdis Vandvik

**Affiliations:** ^1^ Department of Biological Sciences University of Bergen Bergen Norway; ^2^ Bjerknes Centre for Climate Research University of Bergen Bergen Norway; ^3^ Norwegian Institute for Nature Research Bergen Norway

**Keywords:** *Carex pallescens*, *Carex pilulifera*, climate change, disturbance, grassland, *Hypericum maculatum*, range shift, resource acquisitive strategy, resource conservative strategy, seedling recruitment, seed‐sowing experiment, *Succisa pratensis*, *Veronica officinalis*, *Viola canina*, warming

## Abstract

One of the ways in which plants are responding to climate change is by shifting their ranges to higher elevations. Early life‐history stages are major bottlenecks for species' range shifts, and variation in seedling emergence and establishment success can therefore be important determinants of species' ability to establish at higher elevations. Previous studies have found that warming per se tends to not only increase seedling establishment in alpine climates but it also increases plant productivity, which could limit establishment success through increased competition for light. Here we disentangle the relative importance of several climate‐related abiotic and biotic factors on sub‐alpine species' seedling emergence and survival in the alpine. Specifically, we test how temperature, precipitation and competition from neighbouring vegetation impacts establishment, and also whether species' functional traits, or strategies impact their ability to colonise alpine locations. We found that our six sub‐alpine study species were all able to recruit from seed in alpine locations under the extant alpine climate, but their emergence was limited by competition from neighbouring vegetation. This indicates that biotic interactions can hinder the range shifts expected as a result of climate warming. Species with a resource conservative strategy had higher emergence in the extant alpine climate than species with a resource acquisitive strategy, and they were largely unaffected by changes in temperature. The resource acquisitive species, in contrast, had faster emergence under warming, especially when they were released from competition from neighbouring vegetation. Our results indicate that competition from the established vegetation is limiting the spread of lowland species into the alpine, and as the climate continues to warm, species with resource acquisitive traits might gain an advantage.

## Graphical abstract

Plants are responding to climate change by shifting their ranges to higher elevations. There is a large variety in how fast and how far species spread, and we do not understand the factors that allow or restrict subalpine species from colonising new alpine locations. To explore the relative importance of abiotic and biotic factors, as well as species‐specific life strategies, on these limitations, we performed a seed transplant experiment with and without experimental warming, crossed with manipulation of biotic interactions, replicated across a precipitation gradient, with three resource conservative and three resource acquisitive subalpine species.

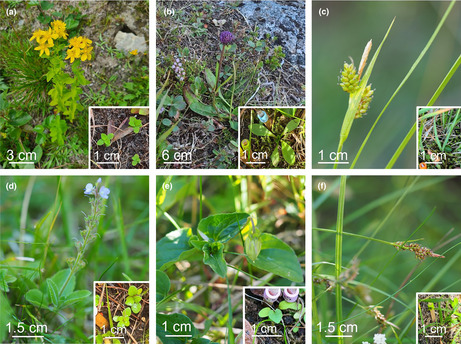

## 1 INTRODUCTION

Climate warming is triggering range shifts of species along elevational gradients (Rumpf et al., 2019), with warming generally happening faster at higher elevations than in adjacent lower elevation areas (Pepin et al., 2015), suggesting increasing rates of colonisation and community change towards higher elevations. Faster upslope colonisation by sub‐alpine species compared to high elevation species (Mamantov et al., 2021), result in altered plant community composition at high elevations and novel interactions between species that have not previously co‐occurred (Alexander et al., 2015). The nature of these novel interactions will depend on the life strategy of the range expanding species, and in particular on adaptations affecting their competitive effects and responses (Funk & Wolf, 2016; Goldberg & Landa, 1991; Kraft et al., 2014). At higher elevations, adverse climatic conditions limit plant life processes, and alpine plant communities are therefore generally characterised by low productivity, by facilitative or neutral plant–plant interactions, and by species with resource conservative life strategies (Körner, 1989; Olsen et al., 2016; Read et al., 2014). At lower elevations, where climate is less limiting, plant communities are generally characterised by higher productivity, by more intense competition for light, and by more functionally diverse communities harbouring species with both resource acquisitive and resource conservative life strategies (Callaway et al., 2002). Accordingly, climate warming is predicted to cause increased productivity, taller vegetation and more standing biomass, and increased competition for light in the alpine (Olsen et al., 2016; Steinbauer et al., 2018). This suggests that climate warming might confer a competitive advantage for resource acquisitive species from the lowlands over the extant species in alpine habitats. However, for sub‐alpine species to establish and spread in alpine locations, they first need to be able to disperse to and successfully colonise alpine locations.

For a majority of plant species, seeds are the only means of long‐distance dispersal, and are therefore a critical part of their ability to shift their ranges (Mondoni et al., 2015; Nathan & Muller‐Landau, 2000). Germinating seeds and young seedlings are vulnerable to numerous abiotic and biotic factors (Baskin & Baskin, 2014; Fenner & Thompson, 2005), and the seedling stage represents, arguably, the most vulnerable part of the life cycle of a plant. Dispersal to and arrival at a new site is no guarantee for establishment, and a species' ability to successfully colonise new sites and shift its range is, therefore, dependent not only on dispersal per se but also on successful seed recruitment in the new sites as the seed must emerge and the seedling must survive (Eriksson, 2000). Both seed dispersal and seedling establishment are considered major bottlenecks in the life history of plants in many ecosystems, and they can therefore limit and delay species from shifting their ranges to higher elevations in response to climate change (Guittar et al., 2020; Shevtsova et al., 2009). Despite the critical importance of seeds and seedlings for species migration and persistence, most studies on climate change in alpine areas focus on the response of adult plants to warming (Briceño et al., 2015; Larson & Funk, 2016). As climate change is expected to greatly impact a host of biotic and abiotic factors that are known to affect regeneration success in the alpine, there is a need for insight into what factors might limit seed germination and seedling recruitment in alpine environments today and under future climates (Mondoni et al., 2015).

In alpine ecosystems, plant development and performance are co‐limited by abiotic factors like low temperatures, short growing seasons and variable moisture availability during the growing season (Shevtsova et al., 2009). Interactions between these limiting factors complicate predictions of climate change impacts. On one hand, climate warming can lead to decreased abiotic stress and extend the period of favourable temperatures for seedling emergence and establishment (Wipf & Rixen, 2010), which would be expected to enhance recruitment speed (i.e. phenological and physiological processes) and success (i.e. number of seedlings that emerge and survive). On the other hand, this increase in temperature can also result in earlier snowmelt and generally higher evaporation losses, which may both increase drought risks later in the growing season (Horton et al., 2006), which could have the opposite effects. In general, lower availability of moisture is related to lower rates of seedling establishment in alpine environments (Forbis, 2003; Meineri et al., 2013). Differences in moisture availability, for instance due to different precipitation regimes, may enhance, moderate, or even shift the direction of the effects of temperature (Töpper et al., 2018), which raises the question of how the net effect of warming and moisture availability on seedling establishment depends on the local climatic context.

Climate change will affect plants not only through direct physiological responses to temperature and precipitation but also indirectly through biotic interactions (Adler et al., 2012). In environments with less abiotic stress, we generally expect more competitive interactions for light (Choler et al., 2001), while towards environments with high abiotic stress we expect facilitative interactions (He et al., 2013). Under warming we, therefore, expect that both the nature and intensity of biotic interactions will change, as demonstrated in studies that report increased intensity of plant–plant competition for light with warming (Meineri et al., 2020; Olsen & Klanderud, 2014). Such indirect, biotic effects of climate warming may be especially important for the vulnerable early life‐history stages of plants (Klanderud et al., 2021; Louthan et al., 2018). Increased competition for light within a denser and taller resident vegetation as the climate warms has been shown to decrease community invasibility and increase competitive effects for light (Klanderud et al., 2017; Meineri et al., 2020; Olsen & Klanderud, 2014). This might give an advantage to colonising species with ‘lowland traits’ such as a resource acquisitive life strategy that may better enable them to compete for light and also exploit low and variable light conditions (Adler et al., 2013).

**FIGURE 1:**
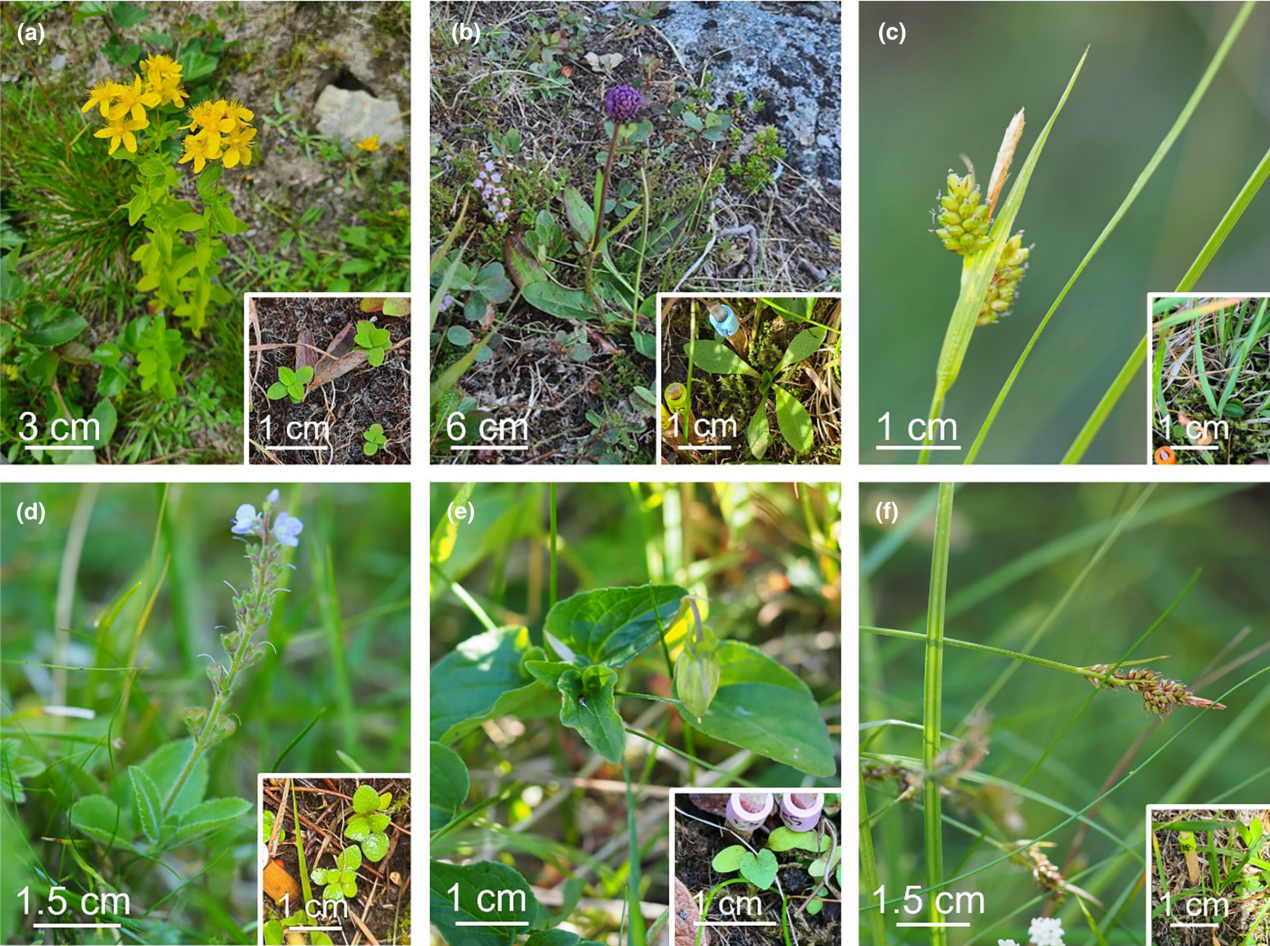
Study species as adults and as seedlings. Resource acquisitive species: (a) *Hypericum maculatum*, (b) *Succisa pratensis*, (c) *Carex pallescens*, resource conservative species: (d) *Veronica officinalis*, (e) *Viola canina*, (f) *Carex pilulifera*. (Photos: adult individuals a, b & seedlings a–f Ingrid Dahle; adult individuals c–f Ragnhild Gya).

In this study, we ask how all these abiotic and biotic factors contribute and interact to limit establishment of sub‐alpine plant species in alpine locations. To answer this question, we performed a factorial seed transplant experiment to assess the interactive effects of climate, biotic interactions and species' resource strategies on subalpine species' seedling recruitment success in the alpine, and replicated this experiment across locations to assess climate context dependencies in these effects. Specifically, to assess the role of temperature, the seeds were sown in alpine locations inside and outside Open Top Chambers (OTCs), that elevated air temperatures by approximately 2°C. This allowed us to assess whether seedling recruitment success of sub‐alpine species in the alpine is temperature limited under current climates, suggesting it will increase under future, warmer climates. To test for effects of biotic interactions, the seeds were sown in plots where the above‐ground vegetation had been removed (bare ground gaps) and in plots with intact vegetation, both inside and outside the OTCs. To test for differences between resource acquisitive and resource conservative species, we selected three species with a resource acquisitive strategy (*Succisa pratensis*, *Hypericum maculatum* and *Carex pallescens*) and three with a resource conservative strategy for the experiment (*Veronica officinalis*, *Viola canina* and *Carex pilulifera*) (see Figure 1). To test for climate context dependencies of these effects, the experiments were replicated at four locations along a precipitation gradient in western Norway, where annual precipitation ranges from 600 to 3000 mm/year. For each of the four alpine study locations, seeds were collected at four nearby sub‐alpine locations differing by approximately 2°C in mean summer temperature (i.e. approximately 400 m elevation difference, www.met.no). This design resulted in a total of 160 plots and 480 species' contrasts (4 locations × 2 warming treatments × 2 biotic treatments × 5 replicate blocks × 2 functional groups × 3 replicate species).

From the argumentation outlined above we expected faster and higher emergence in the experimentally warmed plots relative to the control plots outside the OTCs. As we predicted that warming would shift biotic interactions from facilitative or neutral to more competitive, we expected neighbouring vegetation to have stronger competitive effects in the warmed plots compared to the ambient temperature. Therefore, we expected the effect of vegetation removal (i.e. comparing vegetated to bare ground plots to isolate the biotic effect) to have a neutral to negative effect in the control plots, but to increase seedling emergence and establishment in the warmed plots. We expected the resource acquisitive sub‐alpine species to emerge earlier and establish faster than the resource conservative ones, an effect that we expected would be amplified in the warmer microclimate inside the OTCs. Due to interactions between temperature and moisture effects on seedlings, we expected context dependencies in these patterns along the precipitation gradient, and specifically that the abiotic and biotic effects of warming on recruitment described above should be more pronounced in the wetter locations, where warming mainly increases productivity relative to drier locations, which are more prone to warming‐induced increases in drought risk.

## 2 METHODS

### 2.1 Study locations

**FIGURE 2:**
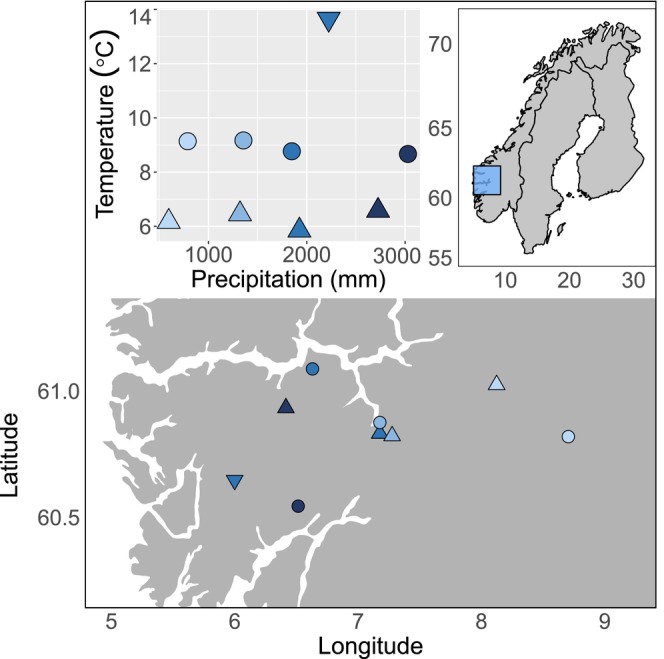
Map of study locations in Western Norway with two levels of temperatures measured with mean of four warmest months (and one extra boreal location, see text) and four levels of precipitation measured in mean annual precipitation. The experiment was conducted in the alpine locations (triangles), while seeds were collected from the sub‐alpine locations (circles) and Bolstadøyri (inverted triangle).

The study was conducted at four alpine locations along a precipitation gradient from the more continental eastern parts to the oceanic western parts of southwest Norway (Figure 2). All locations have a mean temperature of around 6°C during the growing season (four warmest months per year), and annual precipitation ranges from ca. 600 to 3000 mm/year (www.met.no). The locations were selected based on climate data from the normal period 1961–1990 (Klanderud et al., 2015). Seeds were collected from four sub‐alpine locations paired with the alpine study locations, selected to represent the same precipitation gradient, but with a mean temperature of around 8°C during the growing season (Klanderud et al., 2015). The seeds were transplanted to the alpine locations from the paired sub‐alpine locations, or, if seeds were not available from the paired locations, from sub‐alpine locations as similar in precipitation as possible (See Table A1 in Appendix 1 for full overview). For one species (*Succisa pratensis*) we collected seeds at a different location due to low amounts of flowering individuals in the sub‐alpine sites (inverted triangle in Figure 2, and Bolstadøyri in Table 1). To facilitate comparison along the precipitation gradient, the locations were chosen to keep factors other than climate as similar as possible, for example, grazing regime and history, bedrock, vegetation type and structure, slope, and exposure (Table 1). The locations are all grasslands associated with calcareous bedrock (Klanderud et al., 2015).

**TABLE 1:** Overview of locations with mean summer temperature, annual precipitation, bedrock, and coordinates.

Vegetation zone	Location	Latitude	Longitude	Altitude (m.a.s.l.)	Temperature (°C)	Precipitation (mm/year)	Bedrock
Alpine	Skjellingahaugen	60.9335	6.41504	1088	6.58	2725	Marble
	Gudmedalen	60.8328	7.17561	1213	5.85	1925	Rhyolite, Rhyodacite, Dacite
	Låvisdalen	60.8231	7.27596	1097	6.45	1321	Rhyolite, Rhyodacite, Dacite
	Ulvehaugen	61.0243	8.12343	1208	6.17	596	Rhyolite, Rhyodacite, Dacite
Sub‐alpine	Veskre	60.5445	6.51468	797	8.67	3029	(Meta) sandstone, Shale
	Rambera	61.0866	6.63028	769	8.77	1848	Phyllite, Mica schist
	Høgsete	60.8760	7.17666	700	9.17	1356	Phyllite, Mica schist
	Ålrust	60.8203	8.70466	815	9.14	789	(Meta) sandstone, Shale
	Bolstadøyri^a^	60.6477	5.99967	42	13.65	2223	Phyllite

*Note*: Climate data are from 1961 to 1990 normal period. Bedrock data collected from: http://geo.ngu.no/kart/berggrunn/; Climate data collected from: https://www.met.no/. Table modified from Klanderud et al. (2015).

^a^ Bolstadøyri is in the boreal, not sub‐alpine vegetation zone.

### 2.2 Selection of species and seed collection

Six target species for the transplant experiment were selected to represent two contrasting resource strategies, resource conservative and resource acquisitive species (Díaz et al., 2004). The resource conservative species were chosen so that their adult functional trait values resemble those found in the alpine plant communities, whereas the resource acquisitive species were chosen to be more resource acquisitive in terms of at least two of three functional traits (i) higher vegetative height, (ii) lower specific leaf area (SLA), and/or (iii) larger leaf area. These traits were used as they indicate performance with respect to either light interception or growth rate, which have been found to be important for competitive ability for light/space of adult colonising species (Van Kleunen et al., 2010). Generally, the functional traits of adult plants can give an indication of the resource strategy consistent across life‐history stages (Adler et al., 2013; Havrilla et al., 2021; Zhu et al., 2018). This may, however, not always be the case, as ontogenetic shifts in resource strategies between juveniles and adult individuals have been documented in the literature (Dayrell et al., 2018; Mediavilla et al., 2014; Šmilauerová & Šmilauer, 2007). While it would have been optimal to base the species selection for this study on seedling traits, we note that such data are not available for the species in our study system. Also, our study is part of a larger project (Gya, 2022), focusing on the impact of introducing functional novelty to the alpine vegetation, and our study contributes to this research by testing whether recruitment speed and success differed between species with resource conservative and resource acquisitive strategies. Our study species were primarily chosen due to the expected impact of their adult traits on alpine ecosystems, whether they will introduce functional novelty to the system (acquisitive resource strategy) or not (conservative resource strategy), and we tested whether their ability to emerge differed between these strategies.

**FIGURE A1:**
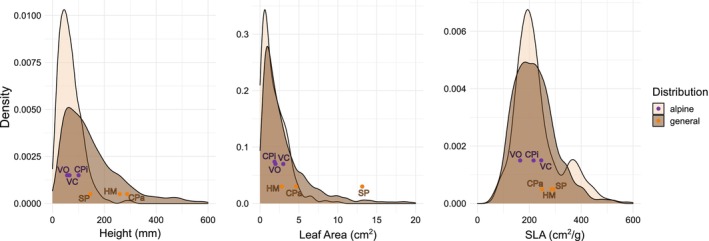
Trait values for our selected sub‐alpine species compared to distribution of traits for alpine species and generalists measured in our study sites (based on data from Gya, 2017). Purple dots mark our resource conservative species (VO—*Veronica officinalis*, VC—*Viola canina*, Cpi—*Carex pilulifera*), while orange dots mark our resource acquisitive species (HM—*Hypericum maculatum*, SP—*Succisa pratensis*, CPa—*Carex pallescens*).

Based on this rationale, and using functional trait data collected from the species in the study sites (Gya, 2017; See Figure A1 in Appendix 2 for full overview), we selected *Veronica officinalis*, *Viola canina* and *Carex pilulifera* as the resource conservative focal species and *Succisa pratensis*, *Hypericum maculatum* and *Carex pallescens* as the resource acquisitive focal species.

For each of these species, as many seeds as possible were collected during the 2020 season, in August and September. Seeds were collected from between 10 and 50 individuals per location depending on the species and how many seeds each individual plant produced. Seeds were stored in room temperature under dry conditions until seeds could be sown in the field.

### 2.3 Experimental design

In each of the locations, we placed five blocks within the target alpine grassland vegetation. Each block consists of a paired design of a plot warmed by an OTC and an ambient climate control. Within each OTC and control, we created two pairs of adjacent 5 × 5 cm^2^ subplots. Within each pair, the right‐hand subplot, when facing the plots up‐slope, was allocated to the intact vegetation treatment and the left‐hand subplot was allocated to the bare ground treatment. Bare ground gaps were created by cutting around the edges and turning the grassland turf in each 5 × 5 cm^2^ plot upside down. One of the pairs of subplots within each OTC or control climate was allocated to the resource conservative species, the other to the resource acquisitive species. This study design resulted in a total of 40 5 × 5 cm^2^ subplots per location (5 blocks × 2 warming treatments × 2 vegetation treatments × 2 life‐history strategies). In the intact vegetation subplots, maximum vegetation height, moss depth and percentage cover of vascular plants and of moss was measured during the peak growing season as indicators of biomass/productivity.

We aimed to sow a minimum of 20 seeds of each species per subplot, as suggested by previous literature (Meineri et al., 2013), but used more when allowed by seed availability. For *Hypericum maculatum*, *Veronica officinalis* and *Carex pallescens*, we sowed 30 seeds per subplot in all locations, for *Viola canina* and *Succisa pratensis* we sowed 20 seeds per plot in all locations, whereas we sowed 25 seeds of *Carex pilulifera* per subplot at only one location due to low seed availability (Skjellingahaugen—the wettest location). This resulted in a total of 10,900 seeds. Seeds were sown in October 2020 to allow the seeds to experience cold stratification in situ during the winter to break any potential dormancy, which is common in boreal species (Baskin & Baskin, 2014; Graae et al., 2022).

During sowing, seeds were scattered evenly on the bare ground in the gaps and onto the intact vegetation. To prevent seeds from being blown or washed away from the bare ground subplots, seeds were pressed down slightly (a few millimetres into the soil surface), but not covered, following Tingstad et al. (2015).

During the 2021 growing season the seedling emergence and survival was surveyed in 2‐week intervals throughout the growing season, from the beginning of June to end of August. At each survey, newly emerged seedlings were marked with numbered toothpicks to enable distinguishing survivors from newly emerged seedlings in subsequent scoring rounds.

### 2.4 Data analysis and hypothesis testing

We used generalised linear mixed effects models (GLMM) to investigate the effects of warming, precipitation context, vegetation and resource strategy on seedling emergence and establishment. All statistical analyses were performed in R (R Core Team, 2020), using the lme4 package (Bates et al., 2015). Our predictor variables were warming treatment (2 factor levels, warmed and ambient), vegetation (2 factor levels, vegetation and bare ground), life strategy (2 factor levels, acquisitive and conservative), and precipitation context (numerical, in meters of annual precipitation, for all location values we subtracted the precipitation value of the driest location, and thus the model intercept represents the driest location). For emergence, we ran models on (i) the total proportion of the sown seeds that emerged, using a binomial error distribution and logit‐link, and on (ii) days to 50% emergence from the start of emergence using a Poisson error distribution with log‐link. For seedling establishment, we ran a model on total proportion of emerged seedlings that survived, using a binomial error distribution and logit‐link. We included location and species in all models as random intercept effects to account for the nested structure of the study design.

As our research questions and specific predictions, as outlined above, concerned interactions between several factors (see below for details), we ran full models including, when possible, a four‐factor interaction to assess how warming, vegetation, life strategy, and precipitation, affected the emergence success. Note that the four‐way interaction was not strictly speaking needed to answer our research questions and test our predictions, as these all deal with three‐way interactions within the proposed fixed‐effects parameterization. Starting from a full factorial approach, we performed a backward elimination to identify the best, most parsimonious model, and used the significance and effect sizes from the final model as tests of specific predictions and to discuss the biological relevance of the different biotic and abiotic factors. This backward selection approach is preferred over a full model approach due to ease of interpretation and avoidance of overfitting. Model suitability with respect to statistical assumptions were visually inspected with normal Q‐Q‐plots and error‐versus‐fitted plots. Using this modelling approach, we addressed the following questions and tested a number of specific predictions:

1. Do species with differing life strategies vary in their seedling emergence and establishment under warming, and do these relationships change along a precipitation gradient?

For *question one* we tested the following predictions: (P1) We expect faster emergence rates and higher final emergence and survival in the experimentally warmed plots compared to the control plots outside the OTCs, (P2) We expect faster emergence rates and higher final emergence and survival towards the wetter locations, and (P3) We expect the resource acquisitive sub‐alpine species to have faster emergence rates and higher final emergence and survival than the resource conservative species, especially in the warmer microclimate inside the OTCs, and towards the wetter locations. Note that this question, if confirmed, will appear as a significant three‐way interaction between the independent variables' life strategy, experimental warming and precipitation.

2 Does the nature and intensity of biotic interactions increase with warming, and do these relationships change along a precipitation gradient?

For *question two* we tested the following predictions: (P4) We expect that emergence will be faster and emergence and survival higher on bare ground compared to vegetated plots in the warmed plots, but that these effects will be weaker or even reversed under ambient climate, and (P5) This effect will be enhanced towards wetter locations. Note that this question, if confirmed, will emerge in the full factorial model as a significant three‐way interaction between the independent variables' biotic interactions, experimental warming and precipitation.

3 Do seedling emergence and establishment for species with differing life strategies vary in their response to biotic interactions under warming?

For *question three* we tested the following prediction: (P6) We expect that resource acquisitive species will tolerate competition from the resident vegetation better, that is, have lower reduction in emergence and survival in vegetated relative to bare ground plots, than resource conservative species, and this difference between life strategies will be amplified in the warmer plots. Note that this question, if confirmed, will emerge in the full factorial model as a significant three‐way interaction between the independent variables' life strategy, biotic interactions, and experimental warming.

## 3 RESULTS

Seedlings of all six sub‐alpine species emerged in the field experiment in the alpine locations. Of the 10,900 seeds sown, 588 developed into seedlings, and 412 survived until the end of the growing season. This gives an overall seed emergence rate of 5.4%, and an overall survival rate of 70%. The species took on average 23 days after snowmelt to reach 50% of total emergence (T50). Overall, the two strategies had comparable T50, but the resource conservative species had higher emergence and survival rates compared to resource acquisitive species (7.4% vs. 3.4% for emergence, and 74% vs. 66% for survival, but with large variation among species).

**FIGURE 3:**
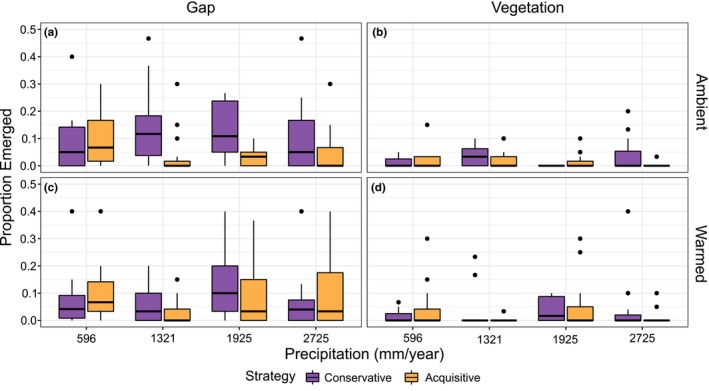
Proportion of seeds emerged as seedlings for resource conservative and resource acquisitive subalpine species along a precipitation gradient (mm/year), under four different treatment combinations; (a) control plot in ambient climate with vegetation removed, (b) control plot in ambient climate with intact vegetation, (c) warmed plot with vegetation removed, and (d) warmed plot with intact vegetation. Y‐axis was set between 0 and 0.5 to better illustrate the data, filtering out an outlier in panel (d) at 1321 mm/year precipitation, a resource conservative species with y = 0.73.

Seedling emergence was highest in bare‐ground gaps, with only about one fifth of all seedlings emerging in intact vegetation (Figure 3). Within gaps, the effect of resource strategy on the emergence success of the seedlings varied with precipitation regime in a unimodal way (Table 2, effects S*P and S*P^2^). At both ends of the precipitation gradient the two strategies had similar emergence rates, but at intermediate levels of precipitation resource conservative species had increased emergence, whereas resource acquisitive species had not (Figure 3a). Experimental warming reduced seedling emergence of conservative species in gaps (Table 2, effect W), but not of acquisitive species (Table 2, effect W*S). In intact vegetation, emergence was too low to show any differences (Figure 3b,d), and thus the mentioned warming effect in gaps were cancelled out in vegetation, according to the model (Table 2, effect W*V).

**TABLE 2:** Model parameters, 95% confidence intervals (CI) and *p*‐values of mixed‐effects models examining the effects of warming (W, levels ‘ambient’ and ‘warming’) and precipitation (P, numeric), resource strategy (S, levels ‘conservative’ and ‘acquisitive’) and biotic interactions from vegetation (V, levels ‘bare ground’ and ‘vegetation’) on seedling emergence rate and timing (T50), and establishment.

Model parameters	Proportion emerged			T50			Proportion survived		
	Estimate	CI	*p*	Estimate	CI	*p*	Estimate	CI	*p*
Intercept	−2.65229	−3.67 to −1.63	**<.001**	2.82084	2.45 to 3.19	**<.001**	0.42160	−0.65 to 1.49	.440
Strategy (S)	0.11802	−1.25 to 1.49	.866	0.18378	−0.32 to 0.69	.475	0.60160	−0.80 to 2.00	.399
Precipitation (P)	0.39682	−0.41 to 1.20	.335	0.50472	0.20 to 0.81	**.001**	2.19017	0.32 to 4.06	**.021**
Precipitation^2^ (P^2^)	−0.14671	−0.45 to 0.16	.351	−0.25088	−0.37 to −0.13	**<.001**	−0.61061	1.32 to 0.10	.093
S * P	−2.00913	−2.82 to 1.20	**<.001**	−0.74213	−1.15 to −0.33	**<.001**	−3.39835	−6.00 to −0.80	**.010**
S * P^2^	0.70270	0.39 to 1.01	**<.001**	0.34928	0.18 to 0.52	**<.001**	1.09979	0.04 to 2.16	**.041**
Vegetation (V)	−1.60760	−2.02 to −1.01	**<.001**	0.16988	−0.06 to 0.40	.143	0.16307	−1.11 to 1.44	.802
V * S	−0.04287	−0.80 to 0.71	.911	0.33240	0.09 to 0.57	**.007**	1.57696	−0.21 to 3.37	.084
V * P	−1.27720	−2.44 to −0.11	**.032**	0.26192	−0.48 to 1.00	.489	−1.64395	−3.55 to 0.26	.090
V * P^2^	0.59062	0.16 to 1.02	**.007**	−0.03701	−0.31 to 0.24	.794	0.58554	−0.17 to 1.34	.127
V * S * P	2.65594	1.04 to 4.29	**.001**	1.07666	0.27 to 1.89	**.009**			
V * S * P^2^	−1.30371	−1.95 to −0.65	**<.001**	−0.76521	−1.11 to −0.42	**<.001**			
Warming (W)	−0.73902	−1.13 to −0.35	**<.001**	0.42599	0.25 to 0.61	**<.001**	0.07154	−0.94 to 1.08	.890
W * S	0.62353	0.26 to 0.99	**<.001**	−0.55957	−0.78 to −0.34	**<.001**	−1.31934	−2.68 to 0.04	.058
W * P	1.29756	0.60 to 2.00	**<.001**	−0.67911	−1.07 to −0.29	**.001**	−2.02640	−4.30 to 0.25	.081
W * P^2^	−0.42457	−0.69 to −0.16	**.002**	0.28764	0.13 to 0.44	**<.001**	0.72358	−0.14 to 1.59	.102
W * S * P				1.82393	1.28 to 2.37	**<.001**	4.15956	0.92 to 7.40	**.012**
W * S * P^2^				−0.79299	−1.02 to −0.57	**<.001**	−1.33726	−2.64 to −0.03	**.045**
W * V	0.43845	0.02 to 0.86	**.041**	−0.55411	−0.79 to −0.32	**<.001**	0.83379	−0.53 to 2.20	.231
W * V * P				0.51547	−0.27 to 1.30	.196			
W * V * P^2^				−0.17094	−0.48 to 0.14	.274			
W * V * S							−2.64774	−4.81 to −0.49	**.016**
W * V * S * P				−2.78818	−3.62 to −1.96	**<.001**			
W * V * S * P^2^				1.49914	1.11 to 1.89	**<.001**			
Random effects									
σ^2^	3.29			0.04			3.29		
τ_00_	0.66 _Species_			0.08 _Species_			0.31 _Species_		
	0.02 _SiteID_			0.00 _SiteID_			0.02 _SiteID_		
ICC	0.01			0.66			0.07		
*N*	4 _SiteID_			4 _SiteID_			4 _SiteID_		
	6 _Species_			6 _Species_			6 _Species_		
Observations	10,900			188			588		
Marginal *R* ^2^/Conditional *R* ^2^	0.192/0.199			0.411/0.799			0.169/0.224		

*Note*: The intercept represents the conservative resource strategy, vegetation gaps, ambient temperature, and precipitation at the driest site. For random effects, we show random effect variance (*σ* ^2^), random intercept variance (*τ*_00_), Interclass‐Correlation Coefficient (ICC) and marginal and conditional *R* ^2^.

Significant values are marked in bold (*p* < .05).

**FIGURE 4:**
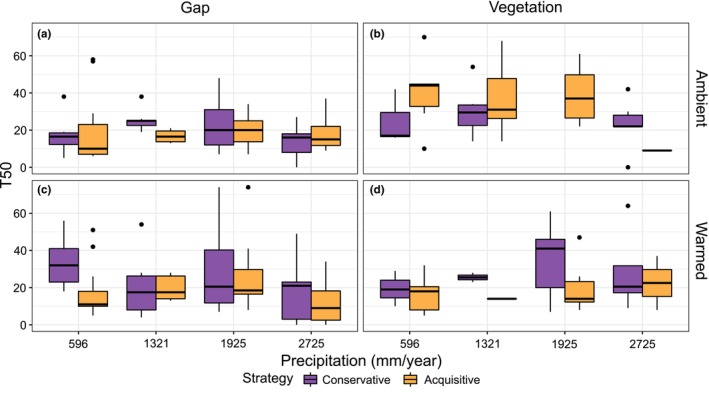
Time to 50% emergence (T50 in days) of resource conservative and resource acquisitive species along a precipitation gradient (mm/year), under four different treatment combinations; (a) control plot in ambient climate with vegetation removed, (b) control plot in ambient climate with intact vegetation, (c) warmed plot with vegetation removed, and (d) warmed plot with intact vegetation.

Seedling emergence was generally slowest in vegetated plots under ambient climate, especially for resource acquisitive species for which it took upwards 30 days for 50% of the seeds to germinate under these treatments compared to 10–20 days in gaps under warming (Figure 4). In contrast, timing of emergence in the conservative species varied less across treatments (Figure 4). In gaps, resource conservative species had a unimodal response to precipitation, emerging faster (i.e. had lower T50) under both very wet and very dry conditions (Table 2, effects P and P^2^). In contrast, emergence timing in the resource acquisitive species appeared largely independent of the precipitation gradient (Figure 4a; Table 2, effects S*P and S*P^2^ counteracting P and P^2^). In intact vegetation, emergence was slower (i.e., increased T50) for the resource acquisitive species compared to the resource conservative ones (Table 2, effect V*S), except at the wettest location (Figure 4b). In gaps, warming slowed down the emergence of conservative species in the extreme ends of the precipitation gradient but promoted earlier emergence in the wettest end for the acquisitive species (Figure 4c; Table 2, effects W to W*S*P^2^). The latter effect (three‐way interaction W*S*P/P^2^ in Table 2) disappeared in intact vegetation (counteracting 4‐way interaction in Table 2). Resource strategy mattered for emergence timing mainly in intact vegetation (Figure 4b,d). The resource conservative species emerged faster than the acquisitive ones under ambient temperatures towards the drier sites (Figure 4b; Table 2, effect V*S, note that lack of data prevents a reliable interpretation of vegetation effects in the wettest sites), whereas the resource acquisitive species emerged faster than the conservative ones under warming at intermediate precipitation (Figure 4d; Table 2, sum of all significant effects).

**FIGURE 5:**
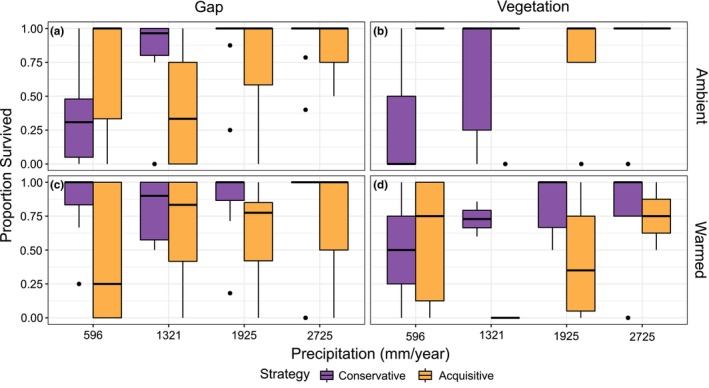
Proportion of emerged seedlings that survived of resource conservative and resource acquisitive species along a precipitation gradient (mm/year), under four different treatment combinations; (a) control plot in ambient climate with vegetation removed, (b) control plot in ambient climate with intact vegetation, (c) warmed plot with vegetation removed, and (d) warmed plot with intact vegetation.

Seedling survival increased with precipitation, especially so in resource conservative species under ambient conditions (Figure 5a,b; Table 2, effect P). In resource acquisitive species, warming reduced the otherwise generally very high levels of survival, and especially so in intact vegetation (Figure 5d; Table 2, effects W*S and W*V*S).

## 4 DISCUSSION

We found that six species that are currently found in sub‐alpine environments can establish from seeds in the alpine environments even under current climatic conditions. While resource conservative species had the overall highest emergence, these species were negatively impacted by warming, especially in bare‐ground gaps. In contrast, the emergence of resource acquisitive species' were facilitated by warming, but this was to some extent counteracted by negative impact on their survival. As expected, competition from neighbouring vegetation reduced seedling recruitment success. We did, however, find that the neighbouring vegetation facilitated survival of the acquisitive species in the ambient alpine climate, but this effect disappeared under warming. The resource conservative species were negatively affected by the presence of vegetation regardless of temperature treatment. We discuss these findings here by first considering the impact of climate warming on emergence (P1) and how the precipitation context impacts this (P2) on the two contrasting life‐strategies (P3). We then consider effects of the neighbouring vegetation under warming (P4) and how this effect changes along our precipitation gradients (P5), before finally considering variation in this competitive effect depending on differing life‐strategies (P6).

### 4.1 Warming increased emergence in acquisitive species, while the conservative species were largely unaffected

We found that species with a resource conservative strategy had an overall higher emergence than resource acquisitive species, and especially so under the current climate. This was not unexpected as the resource conservative species have a strategy similar to the resident alpine vegetation (Gya, 2022). However, the acquisitive species were positively affected by warmer temperatures (confirming P1) in contrast to the resource conservative species, which were largely unaffected. The two life strategies could differ in dormancy‐breaking and germination requirements, as suggested by literature comparing seed germination of strict alpine species with generalist plants that can grow opportunistically in the alpine (Fernández‐Pascual et al., 2021). Also, Milbau et al. (2009) found that warmer summer temperatures increased seedling emergence in some, but not all, species. Other studies that also find a positive relationship between summer temperatures and germination (e.g. Baskin et al., 2002; Graae et al., 2008), do not necessarily consider the functional traits and resource strategy of the species studied. When considering plant species' response to changing temperatures and their resulting range expansion, there is great variation in species traits and responses, although some functional traits are useful predictors (Lynn et al., 2023). While the resource acquisitive species had increased emergence under warming, this came at the cost of a much lower survival rate, suggesting warming is not necessarily facilitating these species' successful colonisation of alpine habitats.

In contrast to our prediction, we found the highest emergence in the middle of our precipitation gradient, a pattern especially prominent among conservative species and in gaps (P2). Increased seedling recruitment towards wetter climates is commonly reported in the literature (e.g. Maestre et al., 2005; Walther et al., 2002); however, most published studies are from much drier regions than our gradient. The few existing studies that do include longer gradients and very high levels of precipitation also find a unimodal relationship with germination peaking at intermediate levels of the precipitation gradient (Tingstad et al., 2015). This pattern could indicate that there is more abiotic stress at not only the dry end of our gradient but also the wet end with high levels of precipitation. When there are high levels of precipitation it can lead to more stress as this shortens the growing season via snowpack, while it can limit access to light via clouds (Lynn et al., 2023).

While acquisitive species might be expected to have a competitive advantage once they have expanded their ranges upward and established into the alpine (Alexander et al., 2018), we found there could be environmental filtering that prevents them from establishing there under current and future climates. This was also supported by our finding that the success of the different strategies also depended on the climatic context of the study location (i.e. the precipitation gradient). The resource acquisitive species emerged faster under warming and in wetter conditions, as expected (P3). This was especially evident in intact vegetation, where emergence under ambient climate was slow. This confirmed our assumption that not only will warming promote seedling emergence in the alpine (Mondoni et al., 2015) but also that species with an acquisitive resource strategy would have a relative advantage in locations where they will have to compete for resources, like light, as their traits and timing of emergence are more variable (Adler et al., 2013).

### 4.2 Competition from neighbouring vegetation decreases emergence, regardless of temperature and precipitation

As expected (P4), we found that gaps in the vegetation enhanced the recruitment success of seedlings. Only one fifth of the seedlings emerged in the intact vegetation, and these seedlings emerged more slowly than seedlings in vegetation gaps—contributing additional evidence that gaps, and thus disturbance, is crucial for seedling recruitment in alpine grasslands (Klanderud et al., 2021; Margreiter et al., 2021; Vandvik et al., 2016). This indicates that neighbouring vegetation had a competitive effect on the establishment success of the species. This was in line with our predictions, as emergence is a vulnerable life stage for plants. The stress‐gradient hypothesis postulates that negative interactions through competition will dominate in warmer and wetter areas with less abiotic stress, while in the colder, drier areas the dominant interactions will shift towards more neutral or even facilitative interactions (Bertness & Callaway, 1994; Olsen et al., 2016).

Contrary to our prediction based on the stress‐gradient hypothesis, however, we found no significant effect of temperatures on the competitive effect of neighbouring vegetation (P4). Although studies have found that OTCs increase biomass and stimulate plant growth in the resident vegetation (Bjorkman et al., 2020; Fazlioglu & Wan, 2021; Wu et al., 2011), we did not find that such an increase in biomass decreased emergence in intact vegetation compared to the ambient alpine temperatures.

Our results also show that precipitation had little to no effect on emergence success in gaps compared to intact vegetation with the presence of potentially competitive neighbours (P5). There was also no significant difference between emergence along the precipitation gradient in the ambient temperatures compared with the warmed temperatures. Whether competition from neighbouring vegetation was increased in the plots with less abiotic stress is, however, difficult to conclude, as the overall low emergence in intact vegetation makes it difficult to detect a significant variation in effects along the climatic gradient.

### 4.3 Neighbouring vegetation negatively impacts resource conservative species, but facilitate resource acquisitive species in colder climates

Neighbouring vegetation reduced seedling emergence of both resource strategies, with no significant differences between strategies, either in the ambient or warmer temperatures (contrary to our expectation P6). However, for the resource acquisitive species, we found that neighbouring vegetation facilitated survival under ambient alpine temperatures, but that this shifted to more competitive interactions under warming where vegetation was more limiting for the acquisitive seedlings' survival. This slight indication of facilitation for the acquisitive species under ambient temperatures compared to competition under warming is what we expected in P6. This is in line with studies that find that at higher elevations, neighbouring vegetation creates more favourable conditions, and the plants growing in intact vegetation are therefore expected to reflect less stress and more acquisitive traits (Reid et al., 2010; van der Merwe et al., 2021). In contrast, the conservative species had lower survival in intact vegetation (i.e. indicative of competitive effects) regardless of temperatures. This was in line with our expectation that resource acquisitive species might have a competitive advantage over the resource conservative species when interacting with the extant alpine vegetation (Adler et al., 2013).

Although it is interesting to note that the survival of the resource acquisitive species was facilitated by neighbouring vegetation under the alpine conditions as they are today, they were not facilitated under warmer temperatures. As productivity increases in warmer temperatures, and abiotic stress decreases, plant–plant interactions are expected to become more competitive (Klanderud et al., 2021; Olsen et al., 2016). Our expectation was that species with more acquisitive traits might do better in the alpine under future warmer climates—which we found to be wrong. Even if alpine areas become generally warmer, these systems will still have more extreme climatic conditions than lower elevations, such as more variation in day and night temperature (Pepin et al., 2015). These conditions are replicated in our experimental warming, as Open Top Chambers elevate temperatures unevenly across time (Gya, 2022; Klein et al., 2005). This could explain why the resource acquisitive species had lower survival rates in the warmer plots, and why they may not be able to shift their ranges and establish in the alpine. Overall, the resource conservative species appeared to have a more similar emergence to what we would expect from the extant alpine species (Meineri et al., 2013), while the acquisitive species emerged opportunistically even though they struggled to persist in the alpine.

### 4.4 Comparison of traits in different life‐history stages—Seedlings to adults

Although there was a clear difference between the seedling recruitment responses of the different resource strategy groups to warming and biotic interactions, it is important to note that there was a large variation between species within both groups. While our results show that adult resource strategies of species affect species' range shift capacities, we know that ontogenetic shifts in ecological strategies can occur, as there can be high plasticity of seedling traits in their early life‐history stages (Dayrell et al., 2018; Havrilla et al., 2021; Larson et al., 2020; Mediavilla et al., 2014). The variation in seedlings traits have been found to reflect a transition from more resource acquisitive traits in the species early development stages, towards more resource conservative traits (Dayrell et al., 2018; Havrilla et al., 2021). Studies have found that traits specifically linked to earlier life history, such as seed mass, can be closely linked to dispersal and establishment success (Gallien et al., 2014; Grotkopp et al., 2002; Hamilton et al., 2005). Explicitly including the traits of seeds and juveniles in future research is therefore critical to understand the role of recruitment as well as the transition from germination to adult individuals in regulating species range shifts under future climates.

## 5 CONCLUDING REMARKS

This study is a contribution to our understanding of how abiotic and biotic factors will impact the recruitment success of sub‐alpine species shifting their range in to the alpine under future climates. More competitive and resource acquisitive range expanding species could potentially have a large impact on the extant alpine ecosystem, more so than species with a more similar resource strategy to the alpine species. Our results show that although sub‐alpine species can establish in the alpine under current climates, there is little evidence that they will thrive, even under the experimentally warmer conditions. The resource conservative species that have more similar traits to the extant alpine species performed better than the more competitive acquisitive species under current climates. The acquisitive species responded more positively to warming, although they still struggled to persist in the alpine even under warming. This indicates that as it gets even warmer more resource competitive species might have an advantage under warmer future climates, and the ensuing shift in resource strategies in the vegetation may have an impact on alpine ecosystem functioning and diversity.

## AUTHOR CONTRIBUTIONS

**Ingrid J. Dahle:** Conceptualization (supporting); data curation (lead); formal analysis (lead); investigation (lead); methodology (lead); visualization (lead); writing – original draft (lead). **Ragnhild Gya:** Conceptualization (supporting); methodology (equal); supervision (equal); writing – review and editing (equal). **Joachim P. Töpper:** Conceptualization (lead); funding acquisition (supporting); methodology (equal); supervision (equal); writing – review and editing (equal). **Vigdis Vandvik:** Conceptualization (lead); funding acquisition (lead); methodology (equal); supervision (equal); writing – review and editing (equal).

## ACKNOWLEDGEMENTS

We thank the senior editor Dr. Chris Foote and the two anonymous reviewers for their thoughtful comments that improved the manuscript in both stages of the registered report. Joshua Lynn, Joseph Gaudard and Egil Dahle assisted with data management and analysis, as well as presenting the results, Sonya Geange and Tordis Dahle provided helpful comments on earlier versions of this manuscript and assisted in the field, and Linn Krüger, Silje Östman, Dagmar Egelkraut, Camilla Zernichow and Tessa Bargman assisted with field site maintenance and fieldwork.

## FUNDING INFORMATION

This project is financially supported by the Norwegian Research Council (funding no. 274712), under the INCLINE project, and by Bjerknes Centre for Climate Research, with personal funding for Ingrid J. Dahle under the Fast Track Initiative.

## CONFLICT OF INTEREST STATEMENT

The authors declare no conflict of interest.

## Data Availability

Data and accompanying script for data cleaning are available on the Open Science Framework (https://osf.io/79vgt/, https://doi.org/10.17605/OSF.IO/79VGT), along with the first stage of the registered report on Authorea (https://doi.org/10.22541/au.164269336.65733239/v1).

## APPENDIX 1

**TABLE A1:** Overview over were seeds that were sown out originated from along our precipitation gradient.

Species\precipitation level	1	2	3	4
*Veronica officinalis*	1	2	2/3	2
*Viola canina*	1	2	1	1/4
*Carex pilulifera*	—	—	—	3
*Succisa pratensis*	3	3	3	3
*Hypericum maculatum*	1	2	2	4
*Carex pallescens*	1	2	3	4

*Note*: Seeds were moved from sub‐alpine locations to alpine locations along the same precipitation gradient with four levels, from dry (level 1) to wet (level 4). Numbers indicate from which level along the precipitation gradient the seeds originated and where they were sown in the alpine. At two locations one species had seeds that were a mix from two source sites (approximately 50% of seeds from each source site).
